# Cancer survivorship programs at the Dana-Farber Cancer Institute

**DOI:** 10.1007/s11764-023-01525-8

**Published:** 2024-01-31

**Authors:** Ann H. Partridge, Alicia Morgans, Lauren P. Knelson, Christopher Recklitis, Larissa Nekhlyudov, Susan N. Chi, Lisa B. Kenney, Lisa Diller, Lynda M. Vrooman

**Affiliations:** 1 Dana-Farber Cancer Institute, 450 Brookline Avenue, Boston, MA 02215 USA; 2 Brigham and Women’s Hospital, Boston, MA USA; 3 Harvard Medical School, Boston, MA USA; 4 Boston Children’s Hospital, Boston, MA USA

**Keywords:** Cancer survivorship, Pediatric oncology, Adult cancer care

## Abstract

**Purpose:**

We sought to present the current status of survivorship programs at Dana-Farber Cancer Institute which include the David B. Perini, Jr. Quality of Life Clinic for survivors of childhood cancer, Stop and Shop Neuro-Oncology Outcomes Clinic for pediatric brain tumor survivors, and Adult Survivorship Program for adult cancer survivors including those diagnosed as adults (age 18 years and older) and adult survivors of childhood cancer, in an effort to share best practices as well as challenges.

**Methods:**

Description of programs and discussion.

**Results:**

Our institutional programs are detailed regarding their history and the multidisciplinary approach and both consultative and long-term care delivery models for pediatric and adult cancer survivors, with the goal of meeting the spectrum of survivorship care needs, from diagnosis and management of long-term effects of cancer-directed therapy and surveillance for subsequent cancer, to healthy lifestyle promotion and psychosocial support. Program investigators conduct research to understand the risks and unmet needs of cancer survivors, and to develop and test interventions to improve care delivery and medical and psychosocial outcomes. There are also educational initiatives detailed.

**Conclusions:**

Survivorship programs at Dana-Farber are designed to optimize care and outcomes for cancer survivors including conducting quality improvement initiatives and research to further understand and meet the clinical needs of the large, heterogenous, and growing population cancer survivors into the future.

**Implications for Cancer Survivors:**

Programs like ours as well as those ongoing and planned aim to improve the comprehensive care of diverse cancer survivors.

## Introduction

The Dana-Farber Cancer Institute (DFCI) was incorporated in Massachusetts in 1951 as a non-profit institution engaged in cancer research and treatment. Initially devoted to pediatric cancer, the DFCI’s mission was broadened in 1969 to include adult cancer research and care. The DFCI is located adjacent to the Brigham and Women’s Hospital (BWH), which provides in-patient services for adult DFCI patients, and to Boston Children’s Hospital (BCH), which provides in-patient services for pediatric DFCI patients. The Dana-Farber Cancer Center was established in 1973 by a National Cancer Institute Comprehensive Cancer Center Core Grant. To include all Harvard Medical School-affiliated institutions including BCH and BWH, it was expanded and re-configured as the Dana-Farber/Harvard Cancer Center (DF/HCC) in 1999. Patients come to the center from across the USA and internationally, although the majority live in the New England region. An EPIC-based electronic health record (EHR) has supported adult patient care delivery since 2015. A Cerner-based EHR system is currently used for pediatric patients with a plan to transition the pediatric EHR to EPIC in 2024.

## Survivorship programs history

*The David B. Perini, Jr. Quality of Life Clinic* was established for pediatric cancer survivors with philanthropic support in 1993. Its mission is to provide and advance excellence in care and to improve the quality of life of childhood cancer survivors through research and education. In 2002, the *Stop and Shop Neuro-Oncology Outcomes Clinic* was added to serve the unique and extensive needs of pediatric brain tumor survivors. In 2004, with grant funding from the LIVESTRONG Foundation and additional support of the Perini Family, the *Adult Survivorship Program* was launched to provide clinical care and support research and educational efforts focused on adult cancer survivors. The Perini Quality of Life Clinic and the Adult Survivorship Program are housed together administratively, philanthropically, and to promote collaborations, under the umbrella of the Perini Family Survivor’s Center. Direct medical services provided to individuals from program clinicians are billed when appropriate and otherwise supported by the institute. Below, the two Pediatric programs and the Adult Survivorship Program are described in detail.

## Pediatric survivorship programs

Pediatric survivorship services are provided in two clinics, the *David B. Perini, Jr. Quality of Life Clinic* and the *Stop and Shop Neuro-Oncology Outcomes Clinic*, which together represent the Pediatric Survivorship Programs. The David B. Perini, Jr. Quality of Life Clinic serves children and adult survivors of the full spectrum of hematologic malignancies and solid tumors diagnosed prior to age 21 years. The Stop and Shop Neuro-Oncology Outcomes Clinic provides care for survivors of pediatric brain tumors. The care delivery approach and structure are similar across both clinics (Table 1).

**Table 1:** Pediatric cancer survivorship services at Dana-Farber Boston Children’s Cancer and Blood Disorders Center

Clinics	Description
David B. Perini, Jr, Quality of Life Clinic	Patients diagnosed age 0–21 years with hematopoietic malignancies and solid tumors, at least 2 years after completion of cancer therapy
Stop and Shop Brain Tumor Clinic	Patients diagnosed age 0–21 years with central nervous system tumors
Staffing	Pediatric oncologists, pediatric and family Advance Practice Nurses, internist, psychologist, Social Work, Oncology Nurse Navigators, care coordinators (administrative)
Subspecialty care	Endocrinology embedded in both clinics
	Dermatology embedded in both clinics
	Neurology and Neuropsychology embedded in Brain Tumor Clinic
	Onco-cardiology at Boston Children’s Hospital
	Other subspecialists at Boston Children’s Hospital
DFCI-based programs for survivors	School Liaison Program
	Fertility preservation -counseling and testing
	Sleep medicine
	Sexual Health Program
	Cancer genetic-risk assessment, counseling, and testing
	Transition Off-Therapy- Care After Pediatric Cancer Treatment
Survivorship planning tools	
EMR	Cerner
Treatment summary and care plan	“ACTS-After Cancer Treatment Summary” web-based, institution developed and supported, customizable survivorship planning tool for both transition off-cancer therapy and long-term follow-up care planning
Community collaborations	
Consortium for New England Childhood Cancer Survivors (CONNECCS)	Founded with mission to improve care of childhood cancer survivors by sharing clinical expertise, research, and education programs among pediatric oncology providers who are consortium members
Research	
Cooperative clinical trials group	Children’s Oncology Group
National cohort	Childhood Cancer Survivorship Study (CCSS)
Institutional prospective cohort	Project REACH
Federally and philanthropically funded	Medical outcomes (cardiomyopathy, fertility, bone health, subsequent cancers, screening practices for late effects, sexual function), psychosocial outcomes (PTSD, suicide, insomnia, sexual health, health care transitions), and health behavioral interventions (insomnia, sexual health, skin cancer prevention)

Both pediatric clinics serve survivors initially treated for cancer within the Dana-Farber/Boston Children’s Cancer and Blood Disorders Center and survivors of childhood cancer treated elsewhere at other institutions. The majority of survivors in the pediatric survivorship clinics are followed on a longitudinal basis for ongoing survivorship care, with a *Longitudinal Model of Care.* In this model, survivors transition from the care of their oncology treatment team to survivorship care. This transition occurs starting at least 2 years from completion of planned cancer-directed therapy, and when after-therapy care focus has shifted from acute toxicity management and frequent surveillance for disease recurrence, to surveillance for and management of long-term and late effects of prior cancer and therapy. In this way, transition timing is tailored to the individual, based on underlying diagnosis and risk of recurrence, with guidelines for transition established between oncology treatment and survivorship leaders. Communication between the DFCI oncology team and the survivorship team at the time point of transition is facilitated by a structured electronic referral form. Survivorship leadership reviews all transfers to survivorships and assigns a primary survivorship provider (described below). Dedicated survivorship care coordinators send welcome to survivorship materials, schedule upcoming visits, and track entry into survivorship care, with the aim of limiting lost-to-follow-up at this transition point. Survivors who were treated for cancer at other institutions, transferring to DFCI survivorship care, are similarly assigned a primary survivorship provider after medical record acquisition and review, and are cared for longitudinally.

Survivors of childhood cancer are followed longitudinally by a primary survivorship provider. Children, adolescents, and young adults are cared for longitudinally by a pediatric oncologist and/or an advanced practice nurse with oncology and survivorship expertise. A medical internist with survivorship expertise serves as the primary survivorship provider for older adult survivors of childhood cancer within the pediatric program. The primary survivorship provider conducts comprehensive survivorship visits as well as visits for targeted survivorship concerns, coordinates care with other subspecialists on the multidisciplinary team, implements follow-up plans, and serves as the point of connection for survivors and primary care provider for survivorship concerns between survivorship visits.

Upon entry into survivorship care, and at regular intervals in their longitudinal follow-up, survivors meet with a team of specialists with expertise in care after cancer therapy for a comprehensive survivorship visit. This visit includes their primary survivorship provider as well as sub-specialty providers as needed (described below). The objectives of a comprehensive survivorship visit are tailored to the needs of the individual patient and include a review of cancer history, continued monitoring for primary cancer recurrence, individualized recommendations for long-term monitoring and management of long-term effects (subsequent cancers, organ dysfunction, growth and development issues), and education regarding risk reduction and health promotion. Imaging and laboratory evaluations are coordinated with comprehensive visits as indicated, for example for cancer recurrence monitoring, subsequent cancer screening, and late effects surveillance. Each comprehensive visit includes review of prior treatment and of the follow-up plan, and treatment summary/survivorship care plan documents are shared with survivors at visits and/or on the patient portal. Detailed visit notes in the EMR are available to survivors through the patient portal and are sent to primary care providers within the EMR or are mailed or faxed if needed.

To facilitate subspecialty consultation and coordinated care, several specialists are embedded within the pediatric survivorship clinics (Table 1). For example, the David B. Perini Clinic includes embedded Endocrinology and Dermatology. The Stop and Shop Neuro-Oncology Outcomes Clinic additionally includes embedded Neurology and Neuropsychology. Psychosocial care clinicians (psychologists and social workers) are embedded within the survivorship clinics and are key members of the care team. Other DFCI-based programs are closely integrated with the survivorship clinics, for example, School Liaison Program, fertility preservation counseling and testing, sleep medicine, and genetic counseling. All survivors are offered consultation with genetic counseling at DFCI, given the increasing awareness of genetic risk for cancer as the underlying etiology of pediatric-onset cancer. Other subspecialty referrals are made by the primary survivorship provider to designated experts within Boston Children’s Hospital (for example, onco-cardiology).

The pediatric survivorship clinics also support a *Consultative Care Model*. Some survivors will continue care with their treating oncology team, particularly in the setting of high risk for primary cancer recurrence or ongoing need for cancer-directed therapies. Oncology providers can refer their oncology patients, at any point in the care trajectory, for survivorship consultation. These consultations are tailored to the individual survivor and include all aspects of a comprehensive survivorship visit as noted above, including the creation of treatment summary and survivorship care plans. In this model, the oncology team implements ongoing follow-up. This model allows survivorship care perspectives to be integrated into ongoing oncology care. The *Consultative Care Model* also includes survivors who were treated for cancer at other institutions, seeking a single comprehensive survivorship consultation, rather than a transfer of survivorship care.

In a long-standing practice, all pediatric survivorship cases are reviewed in a weekly, multi-disciplinary survivorship clinic conference. This format allows the team of multi-disciplinary providers to discuss recommendations for surveillance and management and ensure the provision of comprehensive care.

As pediatric cancer survivors age into adulthood, survivorship care continues either within the above-described pediatric survivorship programs or in the adult survivorship program described below. Primary survivorship providers counsel survivors on transitioning to adult primary care from pediatric primary care. Pediatric cancer survivors who received intensive treatment for rare diseases such as metastatic neuroblastoma, early-onset brain tumors, and infant leukemia are more likely to continue consultation in the pediatric center where that expertise is available. Adult survivors who survived more common pediatric cancers or cancers also seen in adulthood (i.e., childhood leukemia, lymphoma, or sarcoma) are more likely to transition to adult survivorship care. As care is needed for maternal health and management of co-morbidities such as coronary artery disease or development of new adult-onset cancers, childhood cancer survivors also shift to appropriate adult survivor clinics including other subspecialists, and/or adult primary care. Of note, adult survivors of childhood cancer who experience new diagnoses of cancer in adulthood are referred directly to experts in the adult cancer treatment programs.

## Adult Survivorship Program

The *Adult Survivorship Program* aims to enhance adult cancer survivorship care and includes education and research in its mission. It provides general survivorship as well as subspecialty clinical care utilizing a personalized, tailored approach to support patients’ individual needs for follow-up cancer surveillance, general and cancer-specific health prevention, management of late/long-term effects, nutrition, physical activity and well-being strategies, behavior modification support, and coordination of care. The adult survivorship team also conducts innovative patient-centered research focused on understanding and mitigating complications of survivorship (e.g., secondary malignancy biomarkers and risks, fatigue, and sleep), and navigation and support for specific cancer survivor populations (e.g., young breast cancer survivors). The program produces educational materials via a patient-facing website and conducts regular webinars for patients and the public. Finally, we host continuing medical education offerings for oncology clinicians of all types to receive comprehensive education in cancer survivorship.

The Adult Survivorship Program leaders have worked with cancer-site–specific and disease-based leaders at DFCI to create and update specific recommendations for follow-up care to optimize specialized survivorship care for all major types of cancer and treatments. These pathways, which can be tailored to the individual based on national guidelines, are embedded into the survivorship care process using customizable treatment summaries and survivorship care plan templates available in the EPIC EHR. During an initial general adult survivorship visit, regardless of whether the cancer treatment was received at DFCI or elsewhere, the doctor or advanced practice provider (APP, including nurse practitioner or physician assistant) caring for the patient reviews relevant medical records, perform a history and physical exam, and discuss essential elements of the individual’s cancer experience, including treatments received, complications of treatment, and potential late and long-term effects to provide a personalized plan for follow-up. They also provide healthy living tips such as nutrition and exercise guidance, disease-specific counseling on common symptoms, and psychosocial support, as well as subspecialty referrals. Systematic sharing with survivors of relevant clinical studies and supportive care programming has also been piloted in this model to ensure awareness of available supportive care evidence-based interventions and research opportunities [1]. A treatment summary and survivorship care plan (TS/SCP) is provided to the cancer survivor and shared with the patient’s oncology and primary care providers.

**Fig. 1:**
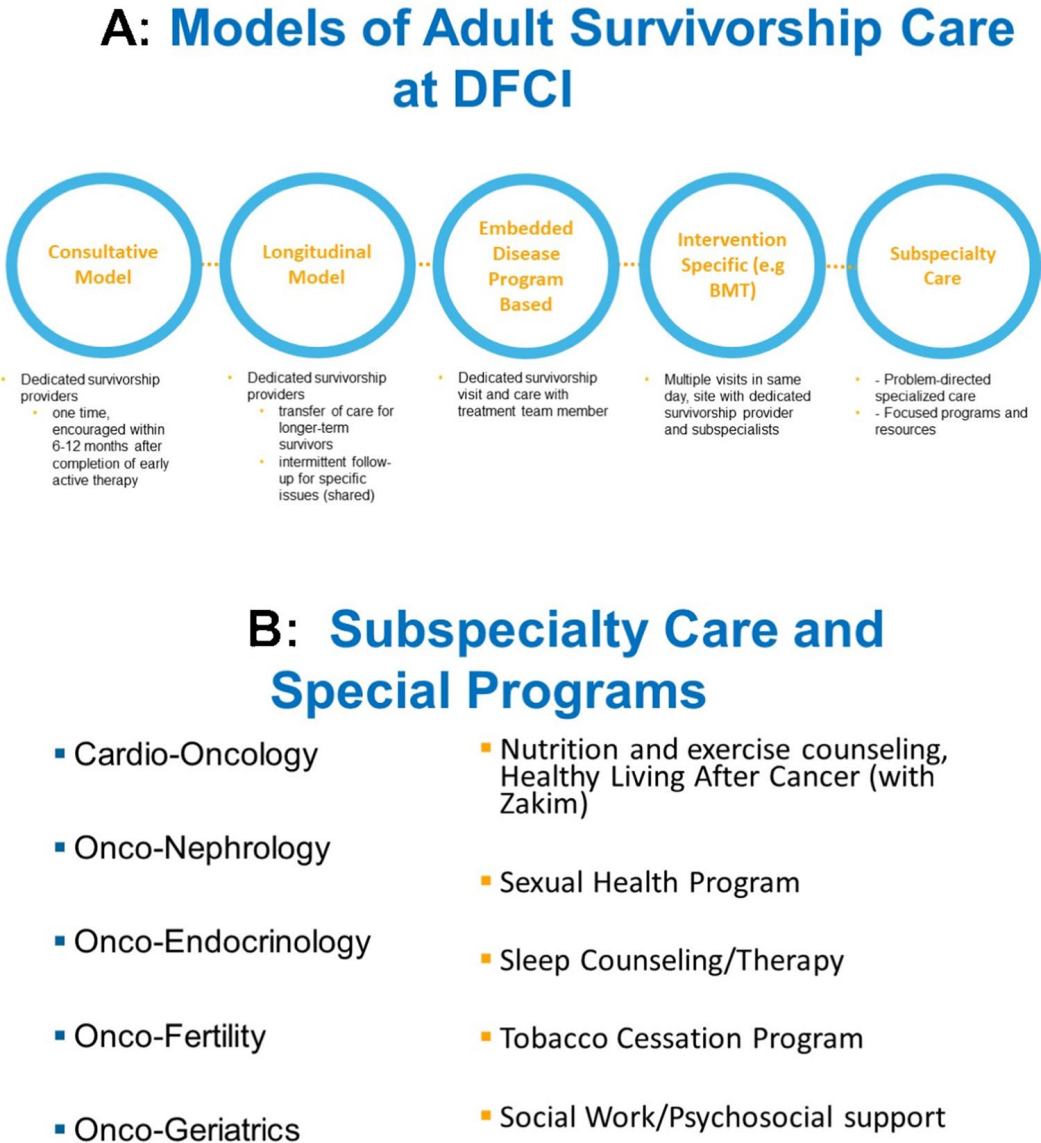
Models of care (**A**) and subspecialty clinics and programs in the Adult Survivorship Program (**B**) at DFCI

Several care delivery models have been developed to implement adult survivorship care. (see Fig. 1, panel A). The *Consultative Care Model* supports a one-time clinical visit for DFCI patients in the stand-alone adult survivorship clinic and is initiated 6 to 12 months after the completion of initial cancer therapy. This visit introduces survivorship care and provides a road map in the form of the treatment summary and survivorship care plan for future care with the expectation that the survivor will follow up with their oncology team, PCP, and other specialists to implement the plan. Two full-time nurse practitioners (NPs) and one part-time physician assistant (PA) trained in adult survivorship care, and an internist, an onco-generalist, provide clinical care in this model. The primary survivorship provider communicates with the patient’s oncology team as needed and provides referrals discussed with the patient. After the visit, adult cancer survivors are advised to continue to follow up with their oncology team for ongoing care and personalized referrals to survivorship subspecialists they received. The *Embedded Survivorship Model* is used for a growing number of disease-based clinics. In this model, the patient’s oncologist or oncology APP schedules and conducts the survivorship visit within 6–12 months after completion of initial therapy and follows the patient through survivorship. This approach was designed to enhance access and reduce stress from transitions by allowing patients to remain in disease clinics with potential future transfer of care to PCP-only or to the survivorship program (longitudinal program, see below).

In the program’s *Longitudinal Model of Care*, patients who are longer-term survivors and no longer receiving any cancer-directed therapy can transfer their care from their primary oncology team to an adult survivorship provider. Communication between the oncology team and the survivorship provider provides continuity and rapid assessments and renewed connection with their disease team clinicians in settings of possible cancer recurrence or questions relevant to their original cancer treatment and risks. The longitudinal model is particularly encouraged for survivors of more than one cancer and those who transfer care to DFCI and who would benefit from follow-up in a cancer center setting but no longer need cancer-directed oncology care.

Given the unique challenges facing survivors of allogeneic bone marrow transplant, a longstanding monthly *Bone Marrow Transplant (BMT) Survivorship Clinic* is a *Specialized Survivorship* Care Model, which is similar to how care is delivered in the pediatric survivorship programs. This program targets patients who are at least 1 year from allogeneic transplant and is led by an oncologist who specializes in BMT. BMT survivors participate in a half-day visit where they receive comprehensive and multidisciplinary follow-up from their oncology team, including a survivorship-focused APP, nutrition, oral medicine, ophthalmology, and social work.

The Adult Survivorship Program at DFCI also houses an array of subspecialty providers and programs to support patients who are receiving disease-directed systemic treatment, no longer receiving therapy, or undergoing long-term maintenance treatment (Figure Panel B). Survivors at DFCI are served throughout the age spectrum. Depending on current age, diagnosis, and treatment history, some adult survivors of childhood cancer are cared for in the DFCI’s Adult Survivorship Program though many are cared for in the Perini Clinic by a dedicated onco-generalist. This flexibility allows for a patient-centered selection of the most appropriate setting for each survivor and is facilitated by the clinic’s location within a cancer center that has a pediatric and adult medical center, with a network of pediatric and adult specialty providers with survivorship expertise.

## Clinical service utilization and Commission on Cancer (CoC) standards

On the pediatric side, the Perini Clinic currently sees over 800 unique patient visits/year, and the Stop and Shop Neuro-Oncology Outcomes Clinic has approximately 300 unique patients/year. The Adult Survivorship Program currently sees more than 4400 patient visits/per year including patients seen by primary survivorship providers and subspecialists. On the adult side, the number of survivorship care plans given over the years has increased from 636 in 2018 to 1528 in 2022.

Reaching all eligible adult survivors to provide a survivorship care plan has been challenging. In light of the evolving Commission on Cancer (CoC) Standard 4.8 of the American College of Surgeons (CoC) metrics (please see www.facs.org for details about this quality program and standards), we have focused our efforts successfully on building our programmatic approach to support the unique and diverse needs of our cancer survivor population. In 2021, we hired a medical director to support our clinical efforts and join the long-standing director to lead the program. We have enhanced existing programs (e.g., onco-cardiology and onco-fertility) and designed and implemented new programs (e.g., a clinical social worker, trained in tobacco cessation, was partnered with the Massachusetts General Hospital Smokefree Support Service to create a Tobacco Cessation program at DFCI). We routinely meet and exceed the CoC standards metrics when we present annually to our institutions’ Cancer Committee (please see www.facs.org for details about this quality program and standards).

## Research, collaborations, and education program components

The pediatric survivorship program engages in a wide range of survivorship research, focused on improving long-term outcomes and the quality of survival, with studies funded by the National Cancer Institute (NCI) and several foundations. Contributions have ranged from descriptive studies of health and psychosocial outcomes [2, 3] and validation studies of patient-reported outcomes [4] to interventions to prevent secondary skin cancers and treat insomnia in survivors [5] (1R21CA261863-01A1; 5R03CA230818-02). As an example of local research efforts, funded by Swim Across America, Project REACH (Research Evaluating After-Cancer Health) is a prospective longitudinal study of outcomes in a cohort of locally treated pediatric and adult cancer survivors that has more than 900 enrolled participants who report on their medical and psychosocial outcomes through regular self-report questionnaires, and has resulted in multiple publications to date focused on issues salient to young survivors including sexual health [6].

On a collaborative research level, DFCI is an original contributing collaborating center for the Childhood Cancer Survivor Study, a multi-institutional, NCI-funded study, of a retrospectively ascertained cohort of childhood cancer survivors diagnosed between 1970 and 1999 as well as siblings of survivors. In addition, pediatric survivorship program investigators lead and engage in cooperative group survivorship research through the Children’s Oncology Group (COG), an NCI-supported clinical trials group, devoted to childhood and adolescent cancer research. For example, a recently completed study of over 350 survivors of childhood high-risk neuroblastoma (ALTE15N2) was developed at the Dana-Farber and the University of Chicago, funded by the St. Baldrick’s Foundation, and conducted as an international cross-sectional study by the COG. This study demonstrated a high burden of toxicity, including growth failure and severe hearing loss among high-risk neuroblastoma survivors. Genetic samples from these patients are currently being submitted for whole-genome sequencing, with the expectation that investigators will identify genetic risk markers for late toxicity. Local and collaborative research efforts continue to aim to improve understanding of and quality of survivorship after childhood cancer.

To further regional collaboration and provide greater reach for services and enhance collaborative research, the *Consortium for New England Childhood Cancer Survivors* (CONNECCS) was formed in 2008. The group membership includes pediatric oncology providers representing all 11 pediatric cancer programs in New England focusing on follow-up care to childhood cancer survivors. CONNECCS holds bi-annual meetings, which include educational sessions addressing important topics in survivorship care and the group works to develop standard guidelines for follow-up where they do not currently exist.

The adult program collaborates with the disease-site-focused adult divisions, subspecialists, and pediatric programs to coordinate care, conduct patient-centered research, and provide education for providers, patients, and the public. Studies initiated by the adult survivorship program specifically include investigations of long-term effects of cancer treatment (e.g., cardiac complications, fatigue, neuropathy, sleep disturbance), biomarkers for risk of second malignancies or other comorbidities, assessment of preferences and concerns of survivor populations and providers, and health care delivery interventions [5, 7–11]. There are long-standing collaborations with other survivorship programs and research efforts including with members of the LIVESTRONG Survivorship Centers of Excellence [12–14]. The work has been funded by the NIH (e.g., U01 CA246648-01A1; U01 CA246659-01A1; R44 CA232905-02A1) and various foundations (e.g., LIVESTRONG, V Foundation, Susan G. Komen, Breast Cancer Research Foundation) and philanthropic support. The adult and pediatric researchers have hosted a number of research retreats together including some open to interested researchers across New England to foster collaborative work.

In terms of education, the DFCI Cancer Survivorship course was designed by program faculty to educate providers about the unique physical and psychosocial effects faced by adult and pediatric cancer survivors and provide guidelines and resources to optimize the delivery and coordination of survivorship care. This course was developed in 2009 as a continuing medical education (CME) program, and it has been institutionally funded with supplemented unrestricted funding by philanthropy and the pharmaceutical industry. Over the years, the course has educated approximately 800 clinicians of all types and researchers, and has received positive evaluations, including the identification of practice-changing goals in caring for cancer survivors. In 2018, to adapt to preferences, expand reach, and serve the needs of the participants who expressed barriers in attending an in-person course, we began to develop an online, interactive program that includes video-recorded lectures by our expert faculty, including case-based learning. The 22-module virtual course is available online to anyone with an interest in enhancing their knowledge on the care of cancer survivors and will be updated every 3 years. (https://cmecatalog.hms.harvard.edu/cancer-survivorship-optimizing-care-and-outcomes). In 2021, a public-facing dedicated survivorship website was created for our program. We subsequently launched a regular webinar series focused on issues of salient importance to cancer survivors and their loved ones, with recordings available on our website (https://adultsurvivorship.dana-farber.org). Teaching sheets that we have created in conjunction with our disease- and subspecialty-based colleagues and utilized in clinic are also available for the public on the website among other resources for cancer survivors.

## Successes, challenges, opportunities, and plans for future

The survivorship programs at DFCI have had tremendous growth and success over the years. This is, in large part, due to our institutional commitment to the programs’ importance in the care of our patients and philanthropic support including from individuals, foundations, and event-based fundraising. Notable advances include leading a robust, multidisciplinary fertility preservation service in collaboration with our partner institutions, starting a tobacco cessation program, and the launching of the patient-facing website to communicate more directly with survivors regarding webinars, survivor resources, educational materials, and research opportunities. We continue to improve our knowledge about the care of cancer survivors and are expanding our clinical research infrastructure to support more survivorship-focused studies and collaborations. We look forward to hosting the biannual *Cancer Center Survivorship Research Forum* in Fall 2025 in Boston. The COVID-19 pandemic has allowed our survivorship program to provide virtual consultations, allowing us to deliver survivorship care to those for whom an in-person visit is a barrier. However, there is uncertainty about whether this model, which we are continuing for select patients now, will be sustainable in the long-term given emerging reimbursement issues [12]. Finally, a tremendous recent opportunity has been the use of oncology nurse navigation to support clinical initiatives, enhance appropriate pairing of patients with support services, and navigate specific vulnerable populations (e.g., young-onset breast [15] and colorectal survivors) to engage in survivorship services more effectively. We are focused on extending survivorship navigation to other patient populations with unique needs, including young adults with other malignancies, the elderly, and underserved populations. We look forward to continuing to grow our programs to continue to meet the needs of cancer survivors in the years to come.
