# Correction to: Cancer survivorship programs at the Dana-Farber Cancer Institute

**DOI:** 10.1007/s11764-024-01588-1

**Published:** 2024-04-12

**Authors:** Ann H. Partridge, Alicia Morgans, Lauren P. Knelson, Christopher Recklitis, Larissa Nekhlyudov, Susan N. Chi, Lisa B. Kenney, Lisa Diller, Lynda M. Vrooman

**Affiliations:** 1 Dana-Farber Cancer Institute, 450 Brookline Avenue, Boston, MA 02215 USA; 2 Brigham and Women’s Hospital, Boston, MA USA; 3 Harvard Medical School, Boston, MA USA; 4 Boston Children’s Hospital, Boston, MA USA

**Correction to: Journal of Cancer Survivorship (2024) 18:34–41**

10.1007/s11764-023-01525-8

The article “Cancer survivorship programs at the Dana-Farber Cancer Institute” written by Ann H. Partridge, Alicia Morgans,Lauren P. Knelson, Christopher Recklitis, Larissa Nekhlyudov, Susan N. Chi, Lisa B. Kenney, Lisa Diller, Lynda M. Vrooman was originally published electronically on the publisher’s internet portal on 31st January 2024 without open access. With the author(s)’ decision to opt for Open Choice the copyright of the article changed on 10th April 2024 to © The Author(s) 2024 and the article is forthwith distributed under a Creative Commons Attribution 4.0 International License, which permits use, sharing, adaptation, [50] distribution and reproduction in any medium or format, as long as you give appropriate credit to the original author(s) and the source, provide a link to the Creative Commons licence, and indicate if changes were made. The images or other third party material in this article are included in the article’s Creative Commons licence, unless indicated otherwise in a credit line to the material. If material is not included in the article’s Creative Commons licence and your intended use is not permitted by statutory regulation or exceeds the permitted use, you will need to obtain permission directly from the copyright holder. To view a copy of this licence, visit http://creativecommons.org/licenses/by/4.0.

Original article has been corrected.

